# How Shall the Degree of Doctor of Medicine Be Conferred?

**Published:** 1879-04

**Authors:** E. Fletcher Ingals

**Affiliations:** 188 Clark St., Chicago


### ARTICLE XVIII.

### HOW SHALL THE DEGREE OF DOCTOR OF MEDICINE BE CONFERRED?

*Messrs. Editors:*—In the call for the convention of medical colleges to be held in Atlanta, May 2d, it is stated that the objects of the convention are "To adopt some uniform system of instruction more in harmony with the requirements of the age," and the following questions are suggested for discussion: "Shall all the colleges require attendance upon three regular courses of lectures during the three separate years ere admitting students to become candidates for the degree of M.D. Is any uniform system possible, and, if so, to what extent is it possible or even desirable at the present time?"

For several years these questions have engaged the attention of the entire profession, but more especially of those engaged in teaching.

Having thought much upon this subject, I accept the invitation which the call extends, for free discussion, and venture to suggest what seems to me a practicable remedy for many of the existing evils due to our present methods of medical training—a remedy which will render it easy to raise the standard for graduation to any desirable grade. With regard to the requirements for graduation, all admit that knowledge is the essential element; but many will disagree as to the best methods of teaching. When or how this knowledge is secured is a matter of little importance providing satisfactory evidence of its possession is required before a degree can be obtained.

We would be glad if the association of colleges could agree to require attendance on three full courses of lectures, and these of nine months each, prior to examination; yet we think the desired result can be obtained without this arrangement.

Practically it matters not where the student gets his information, or how long he is in obtaining it; if he has the requisite knowledge, he should be allowed to graduate; however, as a knowledge of medicine must necessarily come from long and arduous study, probably no injustice would be done by requiring four full years of medical reading and three courses of lectures prior to graduation. In justice to students, we believe the colleges should institute preliminary examinations, or require other satisfactory evidence of a thorough English education, before admission to their classes; for it is clearly unfair to lead a student to believe he can graduate, to waste his time and accept his money and then refuse to give him a degree because he is ignorant of his mother tongue; and certainly, in this age there is no such pressing need for physicians as to excuse the graduation of those not thoroughly qualified. We would not demand a familiarity with the ancient languages, for although their utility is beyond question, they are not essential if the English be thoroughly understood.

If the convention considers the question of requiring attendance on a specified number of courses, the question of fees is likely to arise, and it may be attempted to secure uniformity in this respect. Such an attempt would doubtless lead to discord, for if uniformity in fees were secured, it would militate against the interests of the weaker colleges. The different colleges should remain free to charge as much or as little as they please, excepting the graduation fee; this latter should be substituted by an examination fee of five or ten dollars (sufficient to cover all expenses attending the examination and issuing of degrees), and no part of this amount should in any case be refunded.

In lieu of the graduation fee, colleges might add something to their fees for each course. This arrangement would cost the colleges nothing; it would be just to students and would remove the opportunity which the public now has for charging the colleges with mercenary motives in graduating some members of their classes.

To repeat, we would suggest preliminary examinations, and an increase of the length of time a student shall read medicine before entering as a candidate for graduation; that colleges remain free to charge what they please for a course of lectures, but that the graduation fee be abolished and its place filled by an examination fee; and that every college use the facilities at its disposal as best it may in teaching. But we also suggest, and would be glad to urge upon the thoughtful consideration of the convention, a radical change in the method of conducting the final examinations, which would secure uniformity in the requirements for graduation, and at the same time allow the various colleges, without detriment to any of their own interests or to those of the profession at large, to demand any amount of knowledge they may deem necessary before granting the degree of doctor of medicine.

We would like to see those requirements equal those of the colleges in any other country, but this is a matter which might he left to the wisdom of the general profession if the colleges will unite on a uniform system of joint examinations.

We would suggest the election, each year, of a board of examiners, to consist of thirty members, one-third of whom should be elected by the delegates to the Association of American Medical Colleges, from among the teachers in the various colleges, one-third to be elected by the same body or by the American Medical Association, from the profession at large, and one-third to be appointed by the Surgeon-General of the United States from the medical officers of the army and navy; or what might perhaps be better, the entire board to be appointed by the Surgeon-General.

It should be the duty of this board to prepare lists of questions on the various topics for examination, which should be kept secret until the hour of examination, and on a stated day of each year to furnish these questions, in sealed envelopes, to the various colleges, in which, on stated days, the same date in all of the colleges, the envelopes should be opened before the faculty, members of the local profession and the graduating class. One envelope containing questions for examination upon a specified topic to be opened in this manner each day, and the questions given to the graduating class. Thus, examinations in surgery at ten o'clock a. m., June 1st, or any other date agreed upon; in obstetrics at ten o'clock June 2d, and so on until the examinations are completed, the dates, hours and topics being the same for all the colleges. The student should be allowed three or four hours to write down his answers, due precautions being taken by the faculty and local profession, that he received no written or other aid in his examination. The examination papers might be written with copying ink or pencil, and copies taken of each for future reference.

They should then be sent by express to the Board of Examiners, which should convene on a certain date—for instance, the second day following the last examination—to examine and mark the returns, and issue certificates to the colleges permitting the graduation of all students whose examination average should exceed a certain specified number, say, 70, 80 or 85, the students having been marked on a scale from 1 to 100; 100 being perfect.

If the work of examining the papers should prove too great a task for the Board of Examiners, they might be empowered to select any needed assistants, taking care that the examiners should not know from what students the papers came, and thus avoiding all possibility for the marks being affected by personal friendship or other indirect causes.

No student whose general average fell below the requirements should be allowed to graduate.

Examinations should be held only once a year, and in cases of failure to pass, second examinations should not be granted before the next regular examination.

The adoption of this or some similar method of joint examinations would at once secure a high grade of proficiency in medical graduates throughout the United States, and would thus accomplish an end for which the whole profession is anxious.

By this method no local interests would be affected, each college would still grant its own diplomas, and all could be sure that incompetent men had not been graduated.

It may be objected to this method that such an examination would require much time and expense. Admitting this, would not the ranks of the profession be recruited rapidly enough even then? However, the time probably would not be much more than that now required by the best colleges for their examinations; and even if it should be greater, it could make no possible difference whether the candidate should receive his diploma in ten days or three weeks after the examination, nor would it matter whether he graduated in the latter part of February or June.

The examinations might be held at the most covenient time, and colleges could easily arrange their courses to correspond.

It may be stated that it would be impossible for the board to examine so many papers. This does not seem to me impossible, or even difficult.

Suppose there were fifteen hundred applicants for the degree, and that each of them had been examined on fifteen different topics, we would have 22,500 papers in all, which would be distributed among the different members of the board. Each could easily examine seventy-five papers in one day, making for the whole board 2,250, and ten days of such work would complete the list.

If the examination fee were ten dollars, this would furnish $15,000 to pay the expenses, which would allow $20 per day for fifteen days, or $300 for each member of the board, and would leave a balance of $6,000, out of which to pay the express charges and the cost of diplomas, stationery, etc.

The time would be a matter of no importance; the means would be ample, and the whole could be systematically managed by a permanent secretary.

I have here given an outline of what to me seems a practicable and speedy method of raising the grade of medical education. This is an outline only, but the minutiæ can be easily supplied.

This method would be uniformly just, inasmuch as all students would pass the same examination.

Any method which does not include a joint examination will fall short of the end desired, for however long the course or thorough the teaching, there is always an opportunity for influences to favor the graduation of incompetent men so long as the immediate instructors constitute the graduating board. This is clearly shown by recent complaints concerning the medical schools of Great Britain, and it is not difficult to suggest causes which in certain cases might operate in this way.

Difficulty may arise as to the method of selecting an examining board, from fear that some students would receive beforehand an intimation of the questions to be asked, but it is not reasonable to fear that a board elected annually and pledged to secrecy would be so dishonorable as to adopt any means for favoring individual students or any particular college.

E. FLETCHER INGALS, M.D.

188 CLARK ST., CHICAGO.

